# Update on Diagnosis and Management of Conjunctival Papilloma

**DOI:** 10.1186/s40662-019-0142-5

**Published:** 2019-06-18

**Authors:** Despoina Theotoka, Melina I. Morkin, Anat Galor, Carol L. Karp

**Affiliations:** 1 Department of Ophthalmology, Bascom Palmer Eye Institute, University of Miami Miller School of Medicine, 900 NW 17th Street, Miami, FL 33136 USA; 2 Miami Veterans Administration Medical Center, 1201 NW 16th Street, Miami, 33125 FL USA

**Keywords:** Conjunctival papilloma, Squamous cell papilloma, Optical coherence tomography, Treatment, Interferon, Mitomycin, Surgery, Cryotherapy, Human papilloma virus

## Abstract

Conjunctival papilloma is an acquired benign squamous cell tumor that can present at any age, but most frequently in the third and fourth decades of life. Papillomas have been associated with human papilloma virus (HPV) infection, usually types 6 and 11.

Although histopathological diagnosis remains the gold standard, the advent of newer non-invasive imaging modalities such as optical coherence tomography (OCT) is transforming the way we diagnose and treat ocular surface tumors, including conjunctival papilloma. Management of these lesions can prove a challenge to the treating physician since not all lesions respond to medical and/or surgical therapy and in fact may worsen after surgical manipulation.

In this review, the epidemiology, pathophysiology, clinical characteristics, and diagnosis of conjunctival papilloma including the use of OCT are discussed. Indications, efficacy, and side effects of currently available management options are also reviewed to guide the selection of the best treatment approach.

## Background

The first reported case of conjunctival papilloma dates back to 1883, when Critchett and Juler described a small reddish mass near the inner canthus of a 14-year-old girl presenting with discomfort and slow increase in size for 5 years [1]. Since then, as a result of scientific advances in the understanding of the pathophysiology and management of conjunctival papilloma, both awareness and knowledge about the disease have increased among ophthalmologists and other eye care providers.

Conjunctival papilloma is an acquired benign tumor that arises from the stratified squamous epithelium of the conjunctiva. It can occur in both children and adults, typically with a slow progressive course [2]. This tumor is usually easily identified by clinical examination since the conjunctiva is a readily visible structure, although tarsal lesions may be missed in the absence of eyelid eversion.

Management of conjunctival papilloma is diverse and both medical and surgical approaches have been described. The course of papillomas can be complicated by multiple recurrences, especially in the pediatric population [3]. It is essential for the ophthalmologist to be aware of the tools available to aid in the diagnosis of papilloma and to understand the available medical and surgical therapeutic options.

## Main text

### Epidemiology

Overall, conjunctival papillomas account for 1 to 16% of conjunctival lesions seen in adults and 1 to 10% of the lesions seen in children and adolescents [4–12], with frequencies differing by study population. Conjunctival papillomas are more common in men and occur most often between the ages of 21 and 40 years with a progressive decrease in incidence thereafter [2, 3, 5, 13–16]. This age distribution is similar to that seen in genital human papilloma virus (HPV) infection in sexually active adults [17]. The main reported risk factor for conjunctival papilloma is HPV infection, with studies in the literature reporting HPV detection in 44 to 92% of conjunctival papillomas [2, 15, 18–21].

There is currently no good evidence to support ultraviolet (UV) light, smoking, and immunodeficiency as potential risk factors. A study that took place in Iran showed that papillomas occurred more commonly in the group with sun-exposure < 180 days/year [12]. In regard to human immunodeficiency virus (HIV), while studies have found it to be a risk factor for ocular surface squamous neoplasia (OSSN) [22–24], this has not been shown for papilloma. The only weak suggestion in the literature has been a case of an aggressive papilloma associated with HPV type 33 thought to have enhanced growth by immunodeficiency in an HIV-positive individual [25]. Lastly, although an increased risk of development of genital papillomas has been associated with tobacco use [26], studies have not yet examined the relationship between smoking and conjunctival papilloma.

### HPV association

HPV is a double-stranded circular DNA virus of the papillomavirus family with epithelial tropism that bears oncogenic potential [27]. HPV is classified in five genera, alpha (α)-, beta (β)-, gamma (γ)-, mu (μ)- and nu (ν)- papilloma virus (PV), of which α-PV is typically identified in genital lesions whereas (β)-, (γ)-, (μ)- and (ν)-PV are predominantly isolated in skin lesions [28]. To date, more than 150 HPV types have been identified and classified as low or high risk according to their epidemiological association with cervical cancer [28, 29].

HPV types 6 and 11 are most frequently identified in conjunctival papillomas [2, 3, 15, 16, 18, 19, 21, 30, 31] with a reported frequency ranging from 44.4 to 75.4% and 4.71 to 28% from all lesions, respectively [15, 20, 31]. Types 5b, 13, 16, 20, 23, 33, and 45 have also been detected [25, 31–33]. Additionally, co-infection with different papilloma virus types has been reported as in one study an individual had HPV types 6/11 and 16 identified in the conjunctival papilloma [30]. Low-risk HPV types 6 and 11 are mostly identified in children and adults with conjunctival papillomas [15], while high-risk HPV types 16 and 18 are mostly found in adults with OSSN [34, 35]. This seems to be in agreement with the fact that the majority of condyloma acuminata are associated with low-risk HPV types while high-risk types are mainly associated with uterine cervical intraepithelial neoplasia and cervical cancer [29]. The mode of ocular HPV transmission is thought to vary from vertical transmission from the mother to the infant during delivery to inoculation through ocular contact with contaminated surfaces or hands [30, 36, 37]. Coexistent presence or history of condyloma acuminata, cutaneous and conjunctival papillomas have been reported, implying that HPV infection can simultaneously appear at multiple sites [3, 13, 38, 39]. The presence of HPV has indeed been detected on the fingers of patients (37.5% of females, *n* = 3; 69% of males, *n* = 9) with genital warts [40], however the association between conjunctival and genital/anal papillomas is not clear. In a study of 17 women with HPV-related cervical dysplasia, DNA of HPV 16 was detected in both limbal and cervical swabs by polymerase chain reaction (PCR) in 35% of the patients [6], although no conjunctival papillomas were present [41]. Another study reported that coexistent genital warts and conjunctival papillomas was seen in 4% of patients (3 out of 73) [3].

Multiple groups have confirmed the presence of HPV in squamous cell papilloma with the use of hybrid capture and PCR assays [16]. As mentioned above, HPV has been detected in 44 to 92% of conjunctival papillomas [2, 15, 18–21]. Two of these studies identified HPV in 92 and 81% of 52 and 165 specimens, respectively [15, 30].

The role of HPV in benign and malignant lesions of the conjunctiva is not completely clear as normal conjunctiva had been found to harbor HPV. As above, while one study did not find HPV in normal conjunctivae (*n* = 20) [15], two others did [41, 42]. One detected HPV types 16/18 in 32% of normal specimens (*n* = 19) [42], and the other identified HPV type 16 in 76.5% of patients with normal ocular surface (*n* = 17) [41]. Therefore, HPV may contribute to the development of papilloma lesions but can also be found in normal appearing tissue.

Development of the HPV vaccine and its use in immunization programs are intended to reduce the prevalence of HPV colonization [43]. In the United States, there are 3 prophylactic HPV vaccines available: the bivalent vaccine targets HPV 16 and 18; the quadrivalent targets HPV 6, 11, 16, and 18; and the 9-valent targets HPV 6, 11, 16, 18, 31, 33, 45, 52, and 58 [44]. Prior to the availability of the vaccine, the prevalence of HPV 6, 11, 16, and 18 was 11.5 and 18.5% in females aged 14–19 (*n* = 1363) and 20–24 (*n* = 432) years, respectively [45]. Six years after vaccine availability, prevalence decreased by 64 and 34% in the above mentioned age groups. The same study showed that within the vaccine era, the prevalence of HPV among vaccinated versus unvaccinated females aged 14–24 years was 2.1% versus 16.9%, respectively. While the effect on conjunctival papilloma is unknown, vaccination to include targets of HPV 6 and 11 may decrease the incidence of conjunctival papilloma in the future [46, 47].

### Clinical findings of conjunctival papillomas

Patients can manifest with a broad range of symptoms depending on the size and location of the tumor [3, 48]. Smaller lesions are usually asymptomatic while larger lesions may cause foreign body sensation and dryness due to inadequate eyelid closure and chronic mucus production. In addition, patients may present with conjunctival hemorrhages and/or cosmetic disturbance [3, 49]. Visual impairment can occur with extensive papillomas and, in children, amblyopia can develop if the visual axis is blocked [50]. Conjunctival papillomas can rarely cause complete obstruction of the canalicular and nasolacrimal duct and invade the nasolacrimal sac, with resultant epiphora, bloody tears, and epistaxis [51–53].

**Fig. 1:**
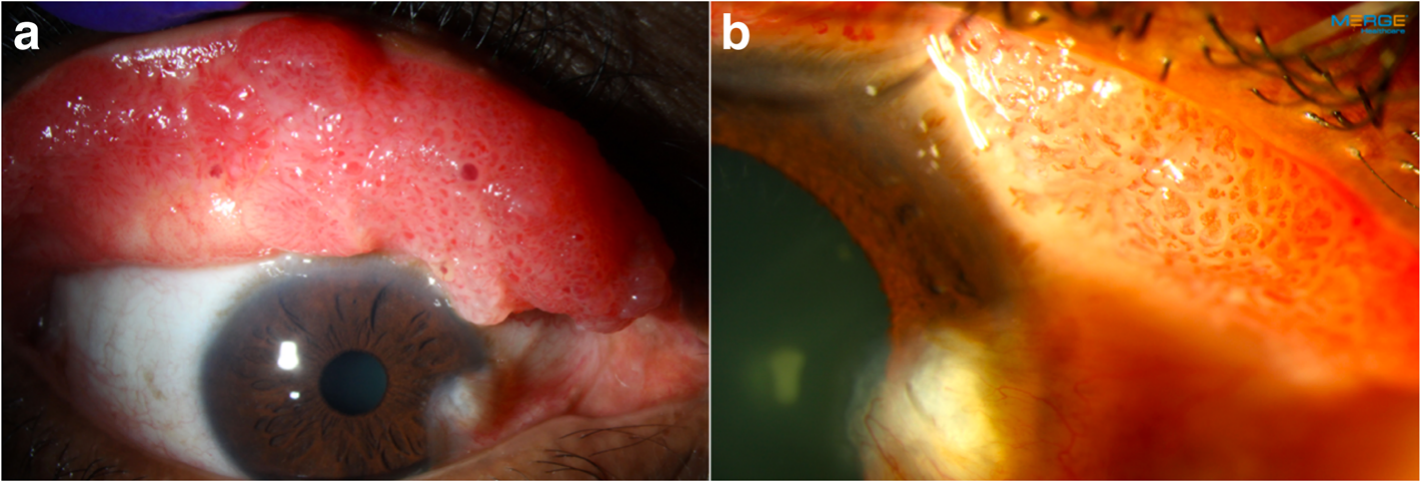
Extensive tarsal conjunctival papilloma. **a**. Slit lamp photograph of a 50-year-old white male with a confluent papillomatous lesion seen with eyelid eversion. **b.** Classic vascular fronds or finger-like projections are easily visualized under the transparent epithelium

Morphologically, conjunctival papillomas can have an exophytic (sessile or pedunculated), mixed, or inverted growth pattern. They are usually characterized by numerous fronds or finger-like projections of epithelium which surrounds a core of highly vascular connective tissue (Fig. 1a and b). The underlying vessels are often seen as multiple “hair pin” vascular loops [54].

Papillomas can present unilaterally or bilaterally, and can be solitary or multi-focal (Fig. 1a) [55]. Lesions in children are often larger than those of adults and are more likely to be multi-focal. These multi-focal lesions can coalesce to form a massive papilloma [3]. In adults, papillomas generally present as solitary, pedunculated tumors, with an abnormal “hair pin” blood vessel pattern [49, 56]. An important way to differentiate papilloma from OSSN is to lift the edge of the papilloma to look for a pedicle, whose presence is almost pathognomonic for papilloma. On the other hand, OSSN is more likely to be part of the conjunctival epithelium, without the ability to lift the edge of the lesion. However, overlap in features can occur. Conjunctival papilloma can be pigmented in darker skinned individuals [57].

In terms of location, some groups have identified the caruncle as the most commonly affected site in adults (24 to 43% of lesions) [3, 10, 48], while others have reported the bulbar (42 to 52%) [5, 13] and tarsal conjunctivae (38%) [2] as the most common locations. In children and adolescents, the most common locations are the inferior fornix (27%) [3] and the caruncle (33%) [9]. Overall, papillomas tend to localize on the nasal and inferior portions of the conjunctiva [2, 10] perhaps explained by autoinoculation of HPV by eye rubbing and collection of the virus medially and inferiorly due to the natural tear flow [2, 9].

### Clinicopathological correlation

The exophytic pattern can be sessile or pedunculated, and is usually covered by multi-layered, non-keratinized squamous epithelial cells and a varying number of goblet and acute inflammatory cells [55]. Exophytic lesions occurring over the limbus tend to be sessile and to have an acanthotic squamous epithelium.

Inverted or endophytic papillomas are made of invaginated lobules of proliferating, non-keratinized squamous epithelial cells which contain goblet cells and grow towards the substantia propria of the conjunctiva [55]. The inverted growth pattern, which is quite rare with only a few references in the literature, carries a greater risk for malignant transformation [58]. The different types of papilloma configuration have been identified to co-exist in the same eye [59].

In one study, histological differences were noted by HPV status. HPV-positive papillomas were mainly composed of basaloid cells with intraepithelial goblet cells, had extra-limbal location, and did not exhibit elastosis [31]. On the other hand, HPV-negative papillomas usually did not have goblet cells, exhibited a perilimbal location, and were associated with elastosis and epithelial keratinization. This suggests that HPV may have a specific mechanism of pathogenicity, and that in HPV-negative lesions, other factors such as UV radiation could be precipitating agents. It was hypothesized that perilimbal lesions were more exposed to UV radiation compared to non-limbic areas [31], and solar elastosis was a common finding with UV damage [60]. Naturally, exceptions to these general findings exist and more studies are needed. Furthermore, although koilocytosis (squamous epithelial cells in which the hyperchromatic nucleus is displaced by a large perinuclear vacuole) [61] is an important morphologic finding of HPV infections of the uterine cervix [62]; different authors have shown that it is not a useful finding for HPV detection in conjunctival tissue as its presence can range from 3 to 40% [2, 3].

Benign papillomas may occasionally contain areas of dysplasia which are characterized by the presence of cytologic atypia, epithelial thickening, lack of goblet cells, and mitotic figures extending beyond the basal layer [54]. On examination, inflammation, keratinization, symblepharon formation, and palpebral conjunctival involvement may be seen in individuals with histologic evidence of dysplasia [63]. In addition, sessile rather than pedunculated papillomas are more likely to contain foci of dysplasia [56]. Nevertheless, carcinoma rarely develops from dysplastic conjunctival papilloma [2, 64].

### Differential diagnosis of conjunctival papilloma

Multiple conditions can resemble conjunctival papillomas to some degree, including benign lesions of the surface epithelium (e.g., benign epithelial hyperplasia, epithelial inclusion cyst, keratoacanthoma, and oncocytoma), vascular lesions (e.g., pyogenic granuloma), malignant lesions (e.g., OSSN, sebaceous cell and mucoepidermoid carcinoma, conjunctival lymphomas, and amelanotic melanomas), secondary tumors, and other ocular diseases (e.g., phlyctenular keratoconjunctivitis, and internal hordeolum or chalazion) [49].

### Diagnosis

Patients should be asked about history of ocular surgery and trauma, malignancy, immunodeficiency, presence of genital warts and risk factors for sexually transmitted diseases, genital HPV, vaccination status, immunosuppression, and UV light exposure. In pediatric patients, maternal HPV exposure should be elicited.

Clinical examination aids in tumor categorization and should include eyelid eversion. Lesion characteristics such as basal dimension and thickness, configuration (exophytic, inverted, or mixed), intrinsic vascularity, feeder vessels, presence of pigment, and tumor location should be noted [3]. Baseline and follow-up slit lamp photographic documentation is also helpful and recommended [3, 49].

Tumor palpation is performed during the slit lamp examination using a cotton-tip applicator under topical anesthesia. Most papillomas should be freely mobile over the sclera with the body of the papilloma forming a mushroom shape over a pedicle attached to the conjunctiva. On the other hand, an epithelial lesion that has invaded the underlying connective tissue will feel fastened to the globe [65] and should suggest OSSN, or a sub-epithelial process such as lymphoma and reactive lymphoid hyperplasia. Diffuse and poorly defined lesions should also raise concern for a malignant process such as sebaceous carcinoma (pagetoid spread).

Biopsy for conjunctival papilloma is controversial. On one hand, histopathological analysis showing the previously described characteristics is the gold standard for diagnosis. On the other hand, there is concern that cutting in the area of the lesion can disseminate the virus and lead to new lesions [48, 49]. As such, excisional biopsy is favored over incisional biopsy. Similarly, noninvasive in vivo diagnostic techniques can help in the management of ocular surface lesions, traditionally including impression cytology as well as relatively newer modalities such as anterior segment high resolution optical coherence tomography (HR-OCT) and ultrasound biomicroscopy (UBM) [66].

Impression cytology, a long-standing diagnostic technique first described for conjunctival use in the ‘70s [67], is the non-invasive removal of ocular surface epithelium by adherence to collection devices (cellulose acetate filter paper or biopore membrane) followed by staining with periodic acid-Schiff, hematoxylin-eosin, and/or Papanicolaou, and cytologic analysis [68]. The main advantages are the relatively easy collection of epithelial samples in an outpatient setting, usual good tolerability by patients, preservation of limbal stem cells that can be affected with surgical methods, and the ability to identify histopathological abnormalities such as dysplasia. However, some disadvantages have made impression cytology fall out of favor. Not all pathology laboratories process these samples due to the need for an experienced cytologist. Additionally, the superficial samples do not allow for assessment of invasion or margin involvement.

Impression cytology has yielded positive results in 77 to 97% of biopsy-proven OSSN [69–71]. However, the literature is scarce for its use in the diagnosis of conjunctival papilloma. In one study, the value of impression cytology was compared to tissue histology in the diagnosis of ocular surface neoplasia. Unfortunately, only one out of the 4 histologically confirmed papillomas was positive by cytology [71]. No other studies have reported the diagnostic applicability of this diagnostic technique in conjunctival papilloma and, therefore, no conclusions can be reached at this point.

As anterior segment imaging evolved with improved image quality, easier operation, and expanded applications, non-invasive imaging diagnosis and surveillance have become a reality and increasingly valuable in the management of ocular surface tumors. While no imaging technique is perfect, these tools can aid in the diagnosis and management of conditions such as OSSN, precluding the need for excisional or incisional biopsies [72].

**Fig. 2:**
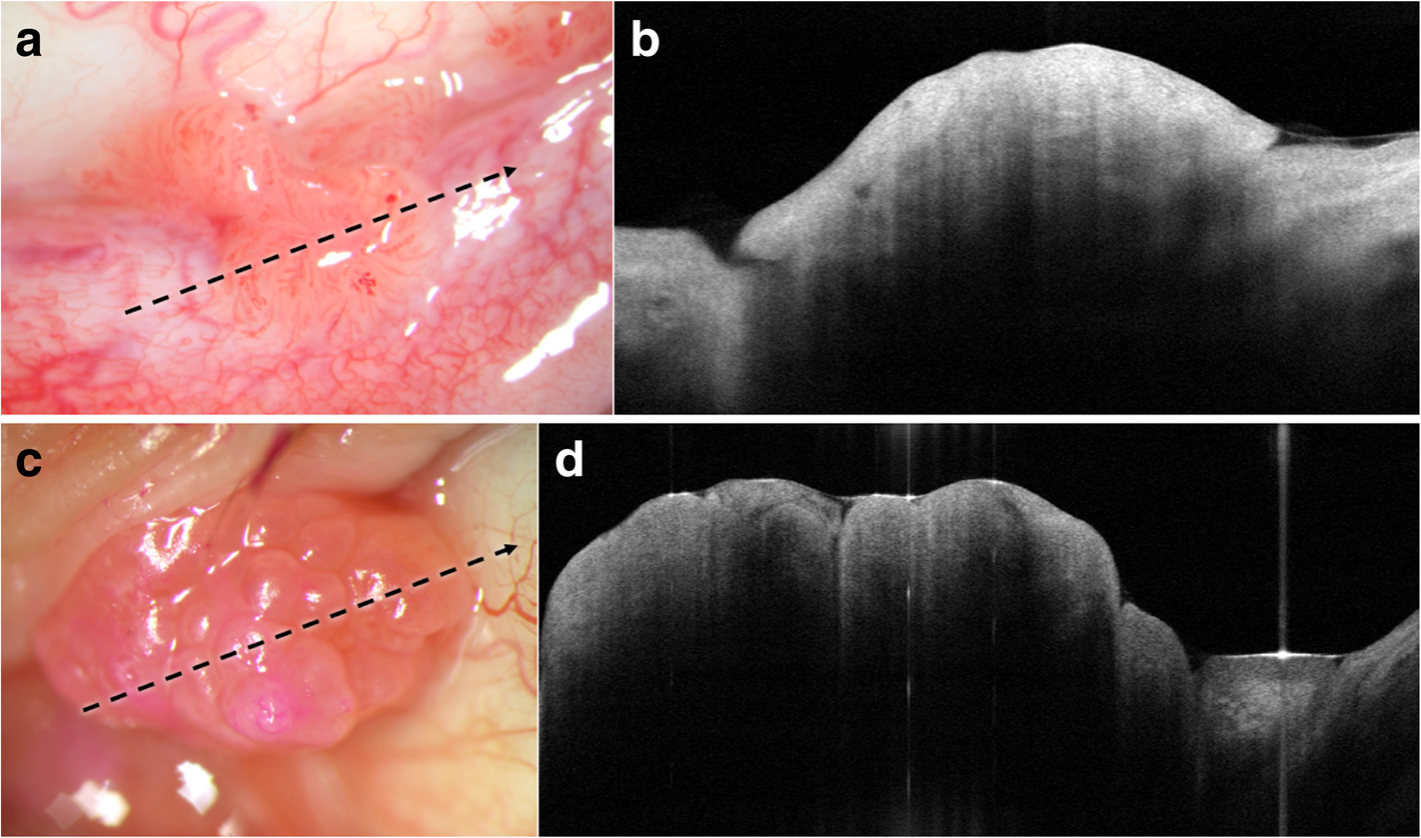
Conjunctival papilloma on high resolution optical coherence tomography (HR-OCT). **a**. Slit lamp photograph of a 51-year-old white male with a sessile tarsal conjunctival papilloma. Direction and location of the HR-OCT scan (black dotted arrow). **b.** HR-OCT shows a well-defined dome-shaped elevation of hyperreflective epithelium. **c.** Slit lamp photograph of a caruncular papilloma in a 66-year-old white male. Direction and location of the HR-OCT scan (black dotted arrow). **d.** An elevated, lobulated, and thickened hyperreflective epithelium is seen on HR-OCT

**Fig. 3:**
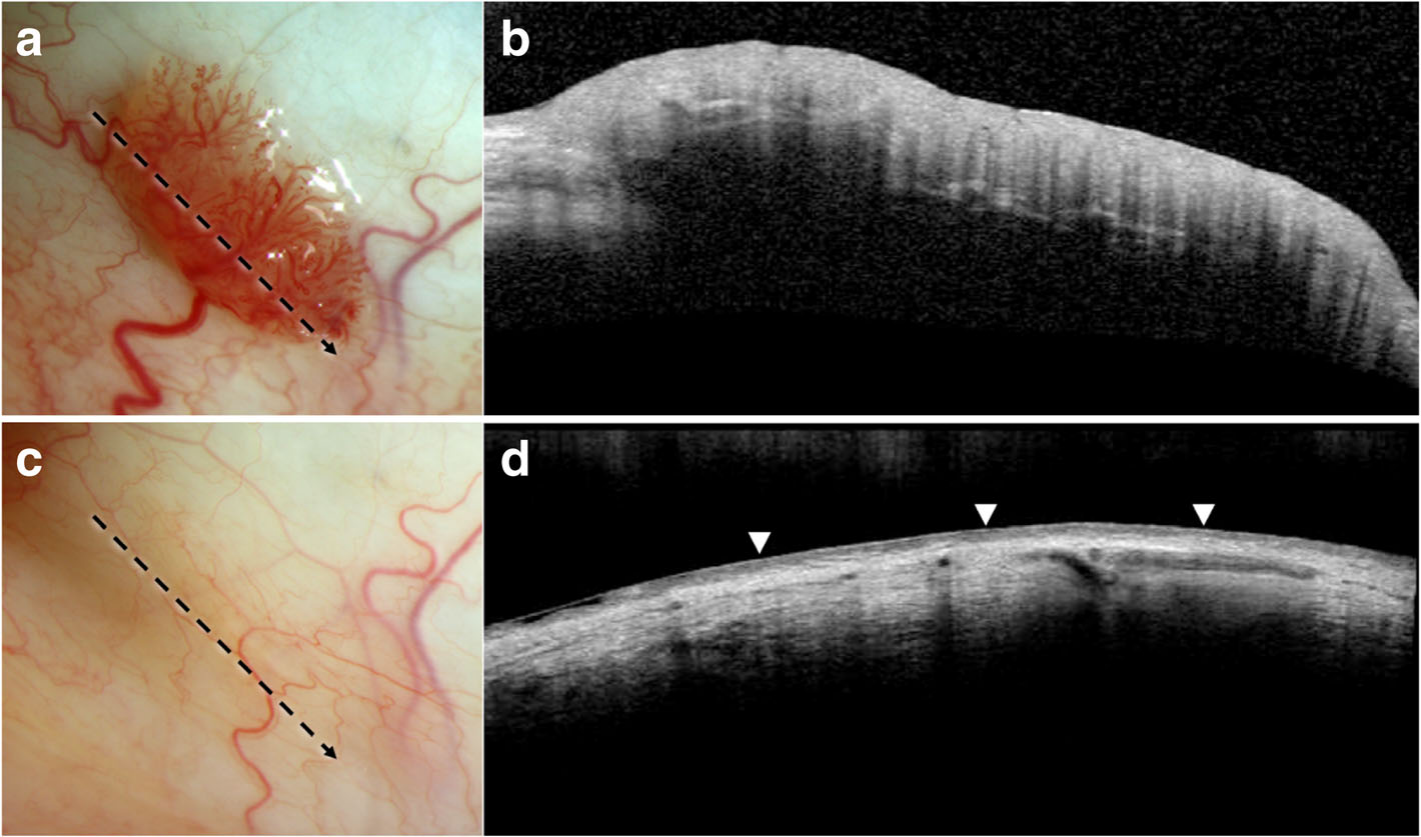
Resolution of conjunctival papilloma on high resolution optical coherence tomography (HR-OCT) with topical interferon (IFN) treatment. **a**. Slit lamp photograph of a 54-year-old white male with a highly vascularized sessile bulbar conjunctival papilloma and a corresponding feeder vessel. Direction and location of the HR-OCT scan (black dotted arrow). **b.** Elevated hyperreflective epithelial layer without abrupt transition to normal epithelium is seen in this papilloma. Hyporeflective lines within the lesion likely represent shadowing from the vessels. **c.** Slit lamp photograph showing complete regression of the lesion after 5 months of topical IFN (1 MIU/ml 4 times daily). Of note, the feeder vessel significantly decreased in caliber as well. Direction and location of the HR-OCT scan (black dotted arrow). **d.** The thickened hyperreflective epithelial mass is resolved on HR-OCT after treatment with IFN. Normal hyporeflective epithelium is indicated with arrowheads

Rapid, non-contact OCT image acquisition does not require a highly skilled operator, and images can be interpreted by experienced as well as novice clinicians [73]. The distinctive features of conjunctival papilloma on HR-OCT have not been previously described. We have found that HR-OCT findings of conjunctival papilloma include a thickened hyperreflective epithelium with or without an abrupt transition to normal epithelium (hyporeflective) and usually displaying a dome-shaped or lobulated configuration in cases of exophytic growth pattern (Fig. 2a-d). The highly vascularized core may also be visualized on HR-OCT (Fig. 3a and b). However, no pathognomonic findings have been identified and studies with larger sample sizes that compare conjunctival papillomas to other ocular surface lesions are needed to determine what other key findings may be gleaned by HR-OCT.

Therapeutically, HR-OCT helps confirm tumor regression by allowing for direct tumor measurement on the scans and comparison of serial pictures. After successful medical therapy or surgical intervention, HR-OCT findings can confirm the normalization of the epithelium (Fig. 3a-d). By detecting subtle lesions not seen on clinical examination, premature termination of treatment may be avoided.

The limitations of HR-OCT include optical shadowing of deeper structures that can occur with large papillomas. In addition, lesions in the fornix and/or caruncle can be difficult to image [74, 75]. While morphological and internal reflectivity changes can be identified with HR-OCT, the resolution cannot yet identify cellular changes of atypia at this time.

UBM is able to penetrate opaque ocular surface lesions, assess presence of invasion, and visualize the posterior border of the tumor at the expense of axial resolution (20 to 50 μm) [76]. As opposed to HR-OCT, UBM requires direct contact with the eye. Additionally, a skilled technician or provider is required to perform the image acquisition and interpretation.

UBM has proven to be helpful in assessing tumor depth and invasion in other ocular surface tumors [77–79], but its utility is limited in noninvasive disease. Furthermore, no studies exist on its specific use for conjunctival papilloma. At this time, UBM remains an additional diagnostic tool to rule out other conditions that may be part of the differential diagnosis of conjunctival papilloma.

### Treatment

In the early twentieth century, wide surgical excision of conjunctival papilloma with cauterization of residual conjunctiva was historically recommended as the best treatment approach [80]. Although excision with cryotherapy is still the most preferred treatment by some [3, 49, 81], postsurgical recurrence is common and can often be dramatically worse than the original lesion [82]. Due to the risks of recurrence and spreading, management of conjunctival papilloma has evolved to include non-surgical treatment modalities in an effort to provide less invasive, more effective and sustained therapies. The advent of topical chemotherapy and immunotherapy has expanded treatment options for conjunctival papilloma, providing not only the possibility of primary non-invasive treatment, but also combined adjuvant treatment along with surgery.

Once the diagnosis and extent of the conjunctival papilloma have been established, there are many factors to consider before deciding on the best treatment modality. The choice of therapy for each individual patient depends on age, systemic comorbidities, location, extension and aggressiveness of the papilloma, ability to comply with medications and/or undergo surgery, and financial constraints.

Considering that slow spontaneous regression can occur in a number of cases, observation and reassurance are reasonable and in fact indicated for small asymptomatic conjunctival lesions [49, 83]. Recurrences can occur after surgical intervention and manipulation of tissue may seed uninvolved areas [49].

A trial of topical steroids can be considered when an inflammatory process (e.g., pyogenic granuloma) is suspected. However, clinical examination and ancillary imaging can typically differentiate the two entities. Conjunctival papilloma generally does not show tumor regression with corticosteroids.

Serial slit lamp photographs should be obtained to monitor for growth or changes in patients, with a frequency of examination between 3 to 6 months depending on the level of concern and progression [49].

### Medical treatment options

#### Interferon alpha-2b

Interferon (IFN) is an endogenous immunomodulatory glycoprotein released by various immune cells with antiviral, antimicrobial and antineoplastic functions [84]. Its anti-oncogenic mechanism of action includes increased immunogenicity by enhancement of dendritic and T-cell function as well as a direct effect on tumor cells through induction of cancerous cell apoptosis [85]. Used in a recombinant form, administration of topical IFN alpha-2b (IFN α-2b) also leads to anti-proliferative and anti-angiogenic effects. IFN has successfully been administered for the treatment of other HPV-related diseases, such as genital papilloma, cervical intraepithelial neoplasia, and OSSN [86, 87]. Furthermore, it can be beneficial in highly vascularized carcinomas, although these mechanisms of action are not well understood [88]. Additionally, the therapeutic effects of IFN have also been attributed to its antiviral properties [84].

Interferon can also be administered subcutaneously for a systemic effect, topically or intralesionally. In terms of systemic use, IFN use for the treatment of conjunctival papilloma was first reported by Lass et al. in 1987 [89]. In this study, interferon alpha-N1 (IFN α-N1) was used intramuscularly as a post-surgical adjuvant treatment in 5 patients with multiple recurrent conjunctival papillomas. Intramuscular injections of IFN α-N1 5 million international units/m^2^ (MIU/m^2^) were administrated daily for 1 month and then 2 or 3 times weekly for another 5 months. Although initially suppressive, recurrence was seen in 3 out of 5 patients upon IFN taper or discontinuation. de Keizer reported using systemic IFN administered subcutaneously 3 times weekly (5 × 10^6^ U) for 6 months for conjunctival papilloma, leading to the shrinkage but not resolution of recurrent conjunctival papillomas in a 38-year old woman [64]. On the other hand, another report of systemic IFN given subcutaneously 3 times weekly for 6 months in a 5-year-old with a 2-year history of HPV 11 PCR-positive conjunctival papilloma reported lesion resolution [90]. In this case, a cutaneous wart appeared 6 months later on the forearm, which was positive for HPV type 27 and was treated successfully with excision and cryotherapy. No additional papilloma recurrences were encountered during the 2-year follow-up period.

Interferon can also be administered directly to the ocular surface, topically or intralesionally, and the choice depends on the location and size of the papilloma [64, 65, 91, 92]. In 2002, Schechter at al. first described the successful use of topical IFN α-2b as primary therapy on one HPV-positive and one HPV-negative conjunctival papilloma [92]. IFN α-2b 1 MIU/ml drops were administered 4 times daily until clinical resolution was achieved, which occurred after 3 months and 6 weeks, respectively, with no recurrence seen after 40 and 18 months of follow-up.

Falco et al. presented another case that responded to primary treatment with topical IFN α-2b (1 MIU/ml 4 times daily) leading to tumor regression in just 2 weeks [65]. Lastly, topical IFN led to resolution in a case of a recalcitrant papilloma with a 4-year history of 12 recurrences (treated with surgical excision, cryotherapy, 5-fluorouracil, systemic IFN-α and CO_2_ laser). However, a new lesion appeared 22 months after the initial regression with IFN and was treated with repeat topical IFN, leading this time to a tumor- free period of 7 years [64].

**Fig. 4:**
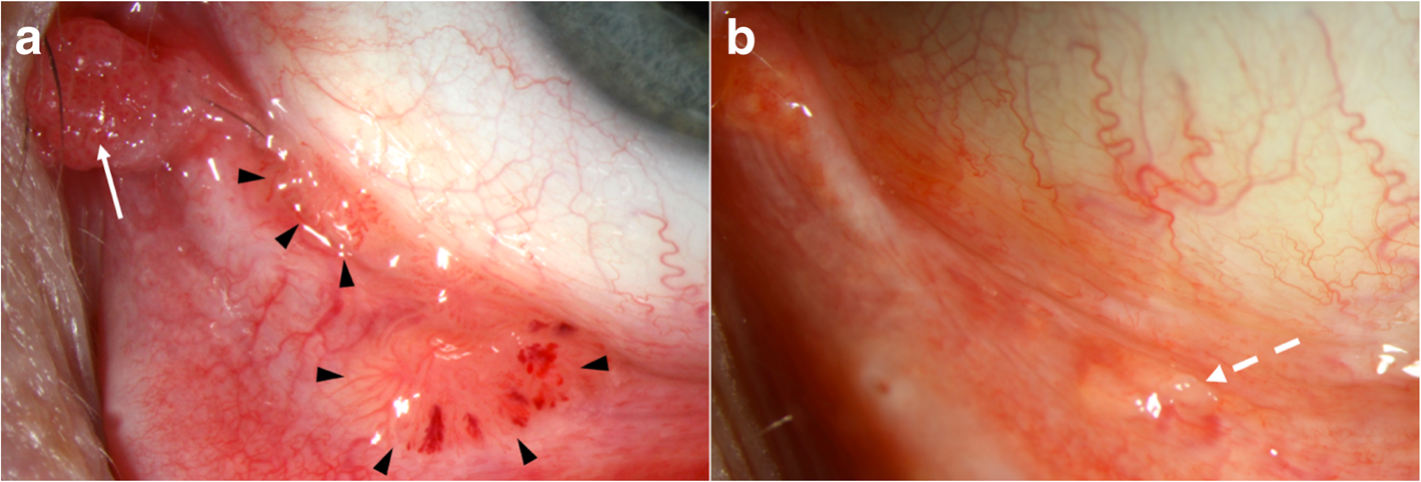
Recurrent multifocal conjunctival papilloma treated with concomitant topical interferon (IFN) and cimetidine. **a**. Sessile (black arrowheads) and pedunculated caruncular (white arrow) papillomas seen on the ocular surface of a 51-year-old white male. Note the spontaneous intralesional hemorrhages in the palpebral lesion. The patient had a history of conjunctival papilloma on the same eye treated with topical IFN 6 years earlier, as well as venereal warts. **b.** After 6 months of topical IFN 1 MIU/ml 4 times daily and 1 month of oral cimetidine 800 mg 3 times daily, the lesion resolved with only follicles remaining (dashed white arrow)

We also favor the use of topical IFN as primary treatment (either alone or followed by surgery if necessary) with a dosage of 1 MIU/ml 4 times daily (Fig. 4a and b**)**.

Topical therapy can be considered for all lesions, but larger tumors may need surgical excision after chemo-debulking. Tumors in a location where prolonged contact with topical medications is difficult (e.g., eyelid margin) generally do not respond as well to topical therapy [91]. Poor compliance and cost concerns also need to be considered. In such cases, as well as in cases of extensive or recurrent papillomas or those that are poorly responsive to topical treatment, intralesional IFN injections can be tried.

In comparison with IFN drops, injections have the advantage of assured compliance. In terms of efficacy, successful primary treatment with intralesional IFN α-2b alone has not been reported [93] (with the exception of pegylated IFN α-2b described below). Instead, a combinational approach for recurrent and resistant papillomas has been reported in many studies [53, 91, 94, 95]. For example, topical IFN eye drops used in combination with subconjunctival and intralesional IFN (0.3 ml of 6 MIU/ml concentration) resulted in tumor regression of a case in 2 weeks; however, the lesion recurred 6 weeks later with treatment cessation [95]. In this case, additional intralesional IFN injections and topical IFN led to tumor regression by the 6-week follow-up. Successful treatment with topical and intralesional injections for recurrent papillomas of the nasolacrimal system has also been documented [53]. Lastly, treatment with IFN drops in a 7-year-old child with 4 papillomas led to regression of a large nasal bulbar conjunctival lesion, but not of those tumors remaining in the lateral canthus and eyelids. Intralesional IFN injections of these non-responding tumors led to complete resolution of 2 out of 3 lesions [91].

Pegylated interferon (PegIFN) α-2b is more potent than IFN α-2b in vitro, while safety and tolerance profiles are similar [96, 97]. Additionally, pegylation of therapeutic proteins is a well-established method for delaying clearance, leading to reduced dosing. PegIFN α-2b injections have been described when all other methods (including 12 surgeries and adjunct treatments) failed in a 23-year-old woman with a confirmed HPV 6 associated conjunctival papilloma [98]. Weekly injections of pegIFN α-2b directly into the papilloma resulted in resolution after 2 months, with no recurrence or adverse effects after 2 years without treatment. The downside of PegIFN is its higher cost compared to IFN [99].

It is not clear why some lesions respond to IFN and others do not. Many factors may play a role including tumor size, location, duration, HPV status, immune status, medication concentration, and frequency of medication administration [90, 91].

In terms of side effects, along with flu-like symptoms and myalgias, patients treated systemically may experience gastrointestinal disturbances (e.g., nausea and vomiting), neutropenia and thrombocytopenia [84]. Furthermore, systemic IFN can cause retinopathy, particularly in patients with vasculopathic disease such as diabetes [100]. Systemic IFN is generally not used in the treatment of conjunctival papilloma given the possibility of local administration with less side effects.

Topical IFN eyedrops are generally gentle on the ocular surface and well tolerated, except for mild conjunctival hyperemia, follicular conjunctivitis, and occasional superficial keratitis [64, 65, 91, 101]. This makes topical IFN attractive in the pediatric population and those with any ocular surface issues. Unfortunately, in the United States, the cost is high, at approximately $600 dollars per month, although it may be much less in other countries. In addition, the drops are used off label, and a compounding pharmacy is required to prepare the formulations [102]. Additional issues to consider include the need for continuous treatment, compliance, and requirement for refrigeration.

Intralesional injections are also well tolerated but do have more significant side effects than topical eyedrops. These include flu-like symptoms such as myalgias and fever seen in a third of the patients [103], which can be ameliorated by administering an oral anti-pyretic (e.g., 1000 mg of oral acetaminophen) at the time of injection and every six hours thereafter. Injections however have the advantage of being commercially available as powder or injection ready solution and no compounding is necessary [102].

#### Mitomycin C

Mitomycin C (MMC) is an alkylating agent derived from the actinobacterium Streptomyces caespitosus that exerts its anti-neoplastic effect by cross-linking DNA [104]. It has been used successfully in OSSN [86]. There is a paucity of data in its use for benign squamous papillomas.

The first report of topical MMC treatment for squamous papilloma was in a patient with diffuse tumor recurrence after 4 surgical excisions with cryotherapy [105]. It was used as an adjunct to excision. Seven days after the fifth excision, a course of MMC 0.02% drops (4 times daily for 2 weeks) was prescribed with the intention to prevent tumor recurrence. No recurrence was noted during a follow-up period of 24 months. Furthermore, MMC has also been used as a primary treatment. In one case, 4 cycles of topical MMC 0.04% (4 times daily; cycles of one week on and one week off) led to complete tumor resolution, and was successfully used as an alternative in an immunocompromised patient who did not respond to topical IFN therapy, possibly due to concurrent tacrolimus use [93]. However, treatment failure with primary topical MMC 0.04% (4 times daily, three 1-week cycles) has also been reported [106].

MMC has a higher frequency of side effects compared with IFN, the most common being ocular discomfort and pain and conjunctival hyperemia [107]. Limbal stem cell deficiency has been reported in 12 to 24% of OSSN patients treated with MMC, particularly with longer treatment course [108]. Other complications include recurrent corneal erosions and keratopathy, corneal perforation, secondary glaucoma and cataract [107] as well as epiphora due to punctal stenosis in up to 14% of cases [109]. Hence, we favor the use of punctal plugs during treatment.

Given this side effect profile, topical MMC is generally considered in cases where IFN has failed or is cost-prohibitive. The cost of MMC is approximately $300 dollars per bottle in the United States and similar to IFN, MMC also needs refrigeration and compounding at a pharmacy and is administered in an off-label manner [102]. It has also been used intraoperatively as an adjunct to surgery, as described below.

#### 5-Fluorouracil

5-Fluorouracil (5-FU) is a pyrimidine analog that blocks DNA and RNA synthesis by inhibiting thymidylate synthase [110]. To our knowledge, there has only been one article in the literature reporting the use of 5-FU as primary and adjuvant treatment of recurrent conjunctival papilloma. They describe a 35-year old woman and a 75-year old man [64]. In the first case, topical 5-FU 1% (4 times daily; the authors did not specify duration) was used after the tenth recurrence of previously benign papillomas with new signs of dysplasia and carcinoma in situ (CIS). However, no improvement was noted, and treatment was discontinued due to several side effects such as corneal and conjunctival erosions. In the second case, post-excisional recurrence of benign papilloma was treated with topical 5-FU 1% 4 times daily. Ectropion and corneal erosion were noted after 4 weeks of treatment and did not resolve despite 5-FU taper. The tumor was then excised and adjuvant 3-month topical treatment with 5-FU 1% combined with retinoid ointment (to prevent corneal side effects) was resumed. Once more, recurrence occurred 3 months later.

**Fig. 5:**
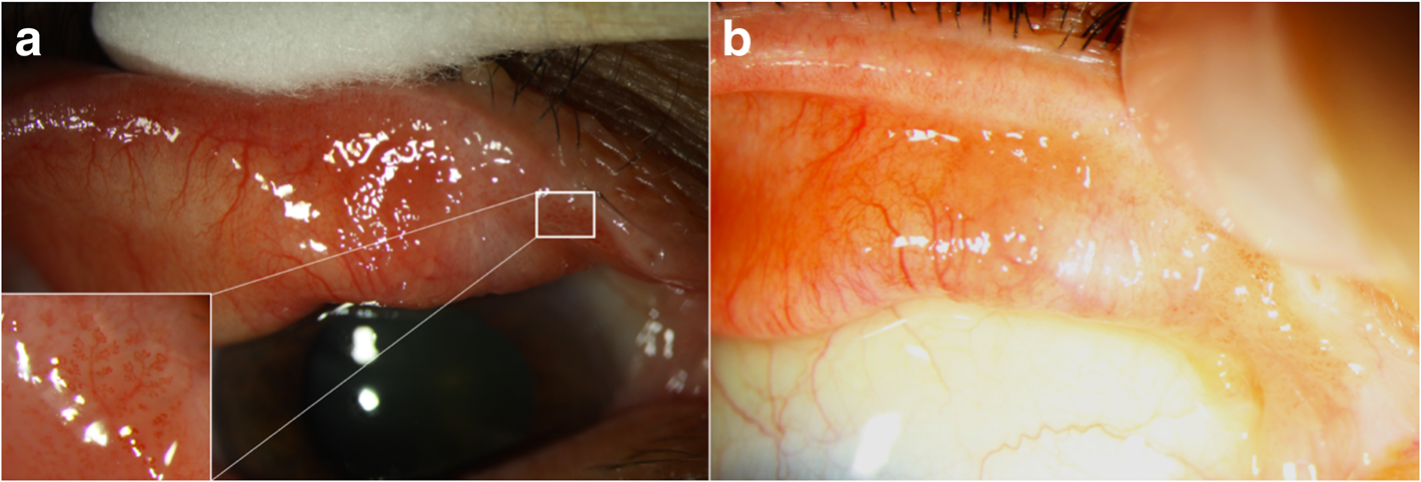
Recurrent conjunctival papilloma treated with 5-fluorouracil (5-FU). **a**. A 78-year-old white female presented with a medially localized recurrence of a previously biopsy-proven poorly defined papilloma covering the entire right upper tarsal conjunctiva and treated with topical interferon 2 years earlier. Inlet: note the fine branching vascularization. **b.** After 4 cycles of 5-FU 1% (1 week on, 3 weeks off), the lesion significantly improved with decreased papillomatous appearance. 2 additional cycles of 5-FU led to tumor resolution. Unfortunately, the lesion recurred 11 months later

We have used cyclical 5-FU 1% (1 week on, 3 weeks off) in a patient with recurrent papillomas 2 years after resolution with topical IFN treatment. After 6 cycles of 5-FU, the tumors resolved (Fig. 5a and b). However, after 11 months, the papilloma recurred.

5-FU has relatively more side effects than IFN but is generally well tolerated. It can sometimes cause ocular pain, conjunctival hyperemia, eyelid edema, superficial keratitis, filamentary keratitis and may rarely cause superficial stromal melting [86, 111, 112]. These symptoms are typically manageable with topical preservative-free tears, a short course of prednisolone, and petroleum jelly on the eyelids. However, in contrast to IFN, it is very affordable (approximately $35 per cycle in the United States) and although it does require compounding, no refrigeration is needed [102].

#### Cimetidine

Cimetidine is an oral histamine H_2_ receptor antagonist mainly used for the management of peptic ulcers. However, at high doses, cimetidine demonstrates immunomodulatory effects evoked by inhibiting suppressor T cell H_2_ receptors and by augmenting delayed-type hypersensitivity responses [113]. Cimetidine has been used as an alternative and safe treatment in pediatric patients with multiple recalcitrant cutaneous warts as well as recurrent respiratory papillomatosis [114, 115]. It is important to note that although three uncontrolled studies showed successful treatment of skin warts with cimetidine, three placebo-controlled, double-blind trials did not validate this clinical effect. A trend toward increased efficacy was seen in younger patients and with higher dosages [116].

**Fig. 6:**
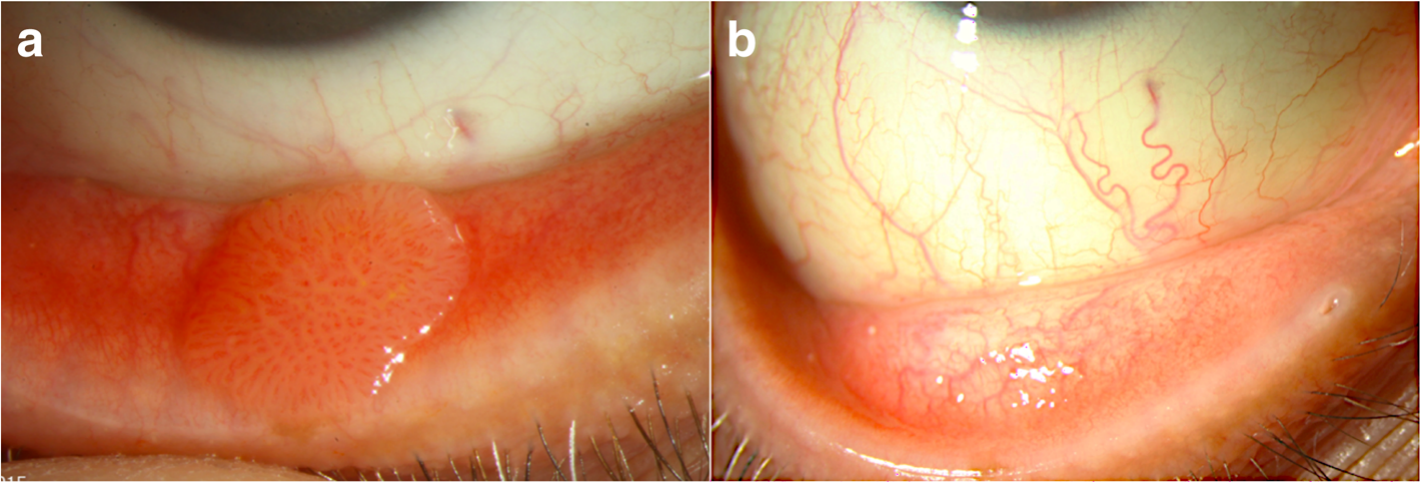
Primary treatment of conjunctival papilloma with cimetidine. **a**. Sessile papilloma located on the tarsal conjunctiva of a 53-year-old white male with history of soft contact lens use and giant papillary conjunctivitis. Note the “hair pin” vessel configuration. **b.** After 3 months of primary treatment with only oral cimetidine (800 mg, 3 times daily), the tumor completely regressed. No recurrence was encountered with a follow up of 16 months

In terms of conjunctival papilloma, cimetidine has been prescribed as a primary treatment, but there is a paucity of publications supporting its use with variable and unpredictable response. One report described dramatic tumor regression noted after 4 months of oral treatment (30 mg/kg/day) in an 11-year old boy when previous therapeutic modalities (excisional biopsy, cryotherapy and topical MMC 0.04%) did not lead to tumor resolution [106]. Of the 6 reported cases in another study, lesions in 2 children did not change with cimetidine therapy, 3 lesions in adults had partial regression and 1 lesion in an adult completely resolved [3]. We have used oral cimetidine successfully (800 mg 3 times daily) alone for tarsal conjunctival papilloma (Fig. 6a and b) and in combination with topical IFN for extensive papillomas (Fig. 4a and b). While we generally place all patients with conjunctival papillomas on oral cimetidine, in our personal experience, only about a mere 10% will respond.

Oral cimetidine has also been used in cases of massive and recalcitrant conjunctival papillomas as presurgical or post-surgical adjuvant therapy to reduce tumor excision burden [117]. Tumor regression was noted after 4 months of oral treatment (30 mg/kg/day) in a 9-year-old patient. Tumor reduction with cimetidine decreased the need for extensive conjunctival resection. This reduced the risk of post-operative conjunctival scarring, symblepharon and ankyloblepharon, and limbal stem cell deficiency [117]. The authors of both studies claim that no systemic or local side effects were encountered [106, 117].

#### Pattern scanning laser photocoagulation

Pattern scanning laser photocoagulation is a fully integrated photocoagulation laser scan system that was proposed for the treatment of conjunctival papilloma as an alternative in low resource settings with limited operating room access [118].

Pattern scanning laser photocoagulation is typically applied after topical anesthesia and toluidine blue 1% instillation to stain areas of high mitotic activity, with treatment administered to an area of 2 mm beyond the tumor edges (20–100 ms, 600–1800 W, spot size 200 μm, 300–1400 shots depending on lesion size and patient tolerability). Mild discomfort was noted by patients during the laser procedure which continued for one to two days but did not require the use of analgesics. No other side effects or complications were noted.

This therapeutic modality was used for primary treatment of 7 eyes of 6 patients with conjunctival papillomas, both pedunculated and sessile. Complete resolution was noted in all 6 patients after an average of 2.3 sessions (range 1 to 6, until resolution via slit lamp biomicroscopy). No recurrences were noted for all patients in a follow-up period ranging from 12 to 15 months.

This technique may be more accessible and cost-effective, however more studies are needed to establish efficacy and cost effectiveness.

#### Photodynamic therapy

Photodynamic therapy is a minimally invasive treatment which uses visible light to activate a photosensitizing drug that can lead to tumor destruction through the action of reactive oxygen species [119]. Photodynamic therapy has been used for the treatment of squamous cell carcinoma and vascular tumors [120, 121] and it has also been described by Kaliki et al. in the treatment of one patient with conjunctival papilloma [3]. The authors report complete regression of a caruncular papilloma after a single photodynamic therapy session. No further reports have been published to date.

#### Surgical treatment

Since its popularization by Shields et al., the “no-touch” wide resection technique has been the traditional method for surgical management of conjunctival lesions with the potential to extend [122]. The procedure is usually performed under monitored anesthesia care with a regional block. Conjunctival forceps and blunt scissors are typically used for excision while care is taken not to touch the tumor with the surgical instruments.

In conjunctival papilloma, surgical excision has been associated with recurrences, which can result in papillomas more severe than preoperatively due to seeding and shedding of viral particles to the surrounding tissue [48, 82, 117]. Identification of microscopic disease beyond the clinically identified lesion is not easily appreciable and thus incomplete excisional biopsy can occur [49]. Furthermore, subsequent surgeries can not only increase the risk of additional recurrences but also lead to further corneal and conjunctival cicatricial changes and even limbal stem cell deficiency in large excisions.

Double freeze-thaw cryotherapy application to the surrounding tissues is known to decrease postsurgical recurrences seen with other ocular surface tumor resections [123, 124] and thus has potential benefit in the treatment of papillomas, although recurrences have been seen even after cryotherapy was added to surgical excision of papilloma [64, 82, 125, 126]. In-office cryotherapy can also be used on small lesions similar to the treatment of warts elsewhere in the body.

**Fig. 7:**
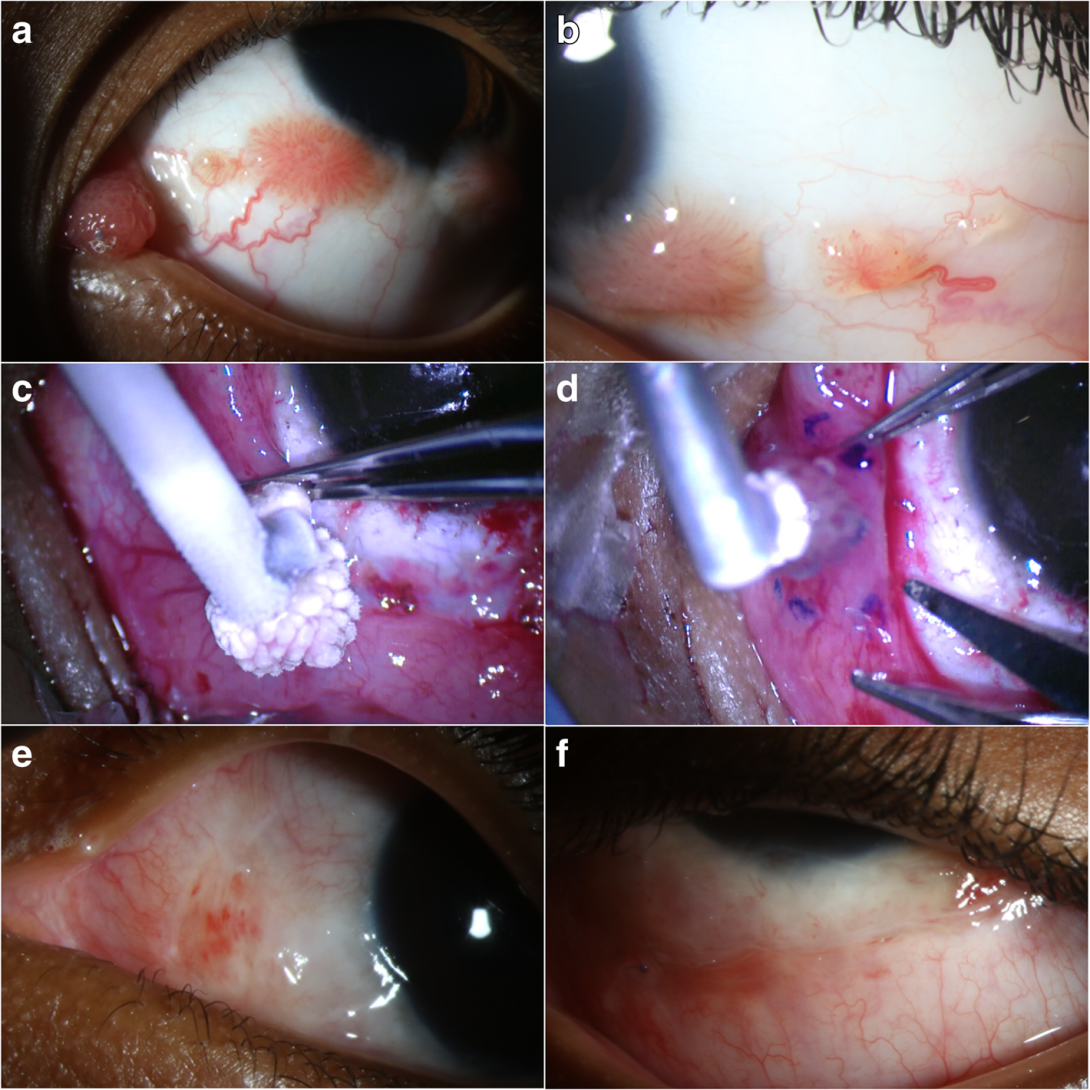
Surgical excision, cryotherapy, and interferon (IFN) α-2b injection for treatment of plical and multiple bulbar conjunctival papillomas. **a**, **b**. A 6-year-old black male presented with multiple papillomatous lesions affecting the caruncle/plica (**a**) and bulbar conjunctiva (**a, b**) in the left eye since age 1. Due to the patient’s age and inability of the mother to instill topical IFN, a decision was made to treat surgically. **c.** Complete cryotherapy was first applied to the papilloma with the intention to minimize viral spreading during subsequent manipulation. **d.** Then, a second cycle of cryotherapy of the entire tumor down to its base was performed. While in frozen state, lifting of the frozen tumor-probe complex allowed for tumor excision with a ‘no touch’ technique and 2 mm margins without the need for forceps. This was followed by cautery and cryotherapy at the borders of the excised conjunctiva and Tenon’s bed, as well as IFN α-2b injection (3 MIU/0.5 ml). An amniotic membrane was sutured to close the remaining conjunctival defect. **e, f.** At one month after surgery, the excision sites were healing well with no visualized papillomas. The amniotic membrane was secure with interrupted vicryl sutures on the tarsal conjunctiva

We recommend a modified double-freeze thaw technique, including a first application of cryotherapy to the entire tumor to kill papillomatous epithelial cells and theoretically minimize viral seeding, followed by a second application down to the tumor base for simultaneous excision. This second complete tumor cryotherapy allows for traction on the lesion without forceps manipulation while excision is performed simultaneously in its frozen state (Fig. 7a-f) [49]. During removal, the base is cauterized. Cryotherapy is also applied to all the conjunctival margins [127].

After tumor removal, either conjunctival undermining and primary closure, autograft from the fellow eye, amniotic membrane or even buccal mucosa transplantation can be used for conjunctival defect coverage [49, 128]. A cultivated conjunctival cell transplant has also been used successfully after removal of multiple conjunctival papillomas in a 10-year old child. According to the authors, this technique allows for earlier epithelization when compared with the traditional use of amniotic membranes, leading to faster healing and decreased incidence of scar formation [129]. However, our preferred method of closure is with cryopreserved human amniotic membrane transplant (AMT), which facilitates rapid epithelialization, spares the remaining conjunctiva and limbal stem cells [130], and is widely available without the need for pre-surgical processing (as with cultivated cell transplants). Fornix deepening sutures and symblepharon rings should be considered in procedures on forniceal and tarsal conjunctiva to prevent fornix shortening [131]. As described below, we favor an injection of 3 MIU/0.5 cc of IFN α-2b at the end of the surgery.

We consider surgery in individuals with large or pedunculated lesions that are symptomatic and in children where amblyopia is a concern [49]. Ultimately, a personalized approach must be taken when deciding between medical management and surgical intervention.

#### Adjuvant medical therapy

MMC has also been administered intraoperatively as an adjuvant agent after surgical excision. Its use intraoperatively for the treatment of conjunctival papilloma was first reported in 1996 [82]. A 5-year-old African American girl experienced recurrent bulbar and palpebral conjunctival papillomas after multiple treatments including cryotherapy, 3 excisions plus cryotherapy, 3 excisions with intraoperative IFN injection and postoperative topical IFN (4 times daily for 2 weeks). Recurrence was noted after each operation with papillomas growing faster and larger. She was successfully managed with a seventh surgical excision and intraoperative application of MMC (0.3 mg/ml for 3 min), which resulted in a disease-free interval of at least 24 months, with development of a small symblepharon as a complication.

Since then, studies have found intraoperative MMC to be effective even in very extensive lesions [50, 132]. Surgical excision with cryotherapy and intraoperative MMC successfully eradicated a diffuse papilloma of the bulbar conjunctiva with mild nuclear atypia encroaching the cornea and a pedunculated papilloma of the caruncle with mild dysplasia [132]. No recurrences were noted for a follow-up period of 10 and 3 years, respectively. Surgical excision with intraoperative MMC were also effective in a child with extensive lesions on the bulbar and tarsal conjunctiva covering most of the palpebral fissure [50]. No complications were reported, and no recurrence was seen for 10 months after surgery.

MMC is applied at concentrations of 0.2 or 0.3 mg/ml via a cellulose sponge, which is held in the area of excision for 2 to 3 min followed by copious irrigation with normal saline [50, 82, 132]. Although severe complications have been reported with the use of intraoperative MMC such as corneal and scleral perforation and endophthalmitis secondary to scleral melting [133, 134], others reported no major side effects when used for the treatment of conjunctival papilloma or other ocular surface lesions [50, 82, 132, 135]. Potential complications may be decreased by avoiding application of MMC directly on the scleral bed [136]. We favor the use a flat piece of plastic (which can be obtained from the disposable packaging of multiple surgical instruments) cut to size of the exposed bare sclera and placed as a platform under the MMC sponges to avoid their direct contact with sclera. This allows treatment of the conjunctival edges with a barrier between the MMC and the sclera.

Topical and/or intraoperative adjuvant immuno- and chemo-therapeutic agents such as IFN α-2b or MMC are administrated in addition to excision in an attempt to reduce the risk of recurrences, especially in resistant and aggressive papillomas. As above, if surgery is needed, our recommended surgical approach consists of a combination of a ‘no-touch’ surgical technique with adjunctive double freeze-thaw cryotherapy and intralesional IFN α-2b (3 MIU/0.5 ml) at the time of surgery. However, there are no randomized control trials comparing the effectiveness of surgical resection with and without adjuvant therapies, and treatment recommendations are mostly based on small or anecdotal reports. A recent publication showed that excisional biopsy, cryotherapy, intralesional IFN and post-operative topical IFN for 3 months was successful in eradicating recurrent, multifocal papillomas in a 2-year old child who did not respond to primary topical IFN drops [137]. No recurrence was noted for 14 months. Other authors also favor a triple approach with complete tumor excision, cryotherapy, and adjunctive oral cimetidine (300–400 mg 3 times daily) and/or topical IFN α-2b for 3 months after surgery [3]. In spite of the combined treatment approach undertaken, recurrences can unfortunately still occur [3, 98, 138].

#### Dinitrochlorobenzene

Dinitrochlorobenzene (DNCB) is a chemical first identified in the study of glutathione-S-transferases and found to cause a type IV hypersensitivity reaction [139]. Its protocol for conjunctival papilloma treatment involves a single topical application of DNCB on the forearm in order to sensitize the body [140]. After application, sensitization is confirmed by the development of a prominent flare at the test site. DNCB can then be applied directly to the conjunctival papilloma, both topically and intralesionally on multiple occasions.

DNCB immunotherapy has been used for the treatment of conjunctival papilloma with mixed results. In 1981, DNCB immunotherapy (topical and subconjunctival injections) was first reported to be successful in a 24-year old man who suffered from a benign papilloma recurrent with atypia and resistant to electrocautery, surgical excision and cryosurgery [126]. The patient improved after 7 applications of DNCB over 8 weeks (50% reduction of the papilloma’s original mass). He was however lost to follow-up and then reappeared with a lesion increased in size. He underwent 6 more DNCB treatments and remained tumor free for a period of 10 months. Similar results were reported 2 years later when DNCB was used as an adjuvant treatment after surgical excision of recurrent papillomas in a 4-year old boy [125]. No recurrence was noted for 8 months. However, the third case report showed no lesion resolution with topical application of DNCB for the treatment of recurrent papillomas (increasing concentrations from 0.1 to 2%) [140]. With respect to side effects, corneal scarring and superficial peripheral vascularity have been noted [125]. The role of DNCB in the treatment of conjunctival papilloma remains, therefore, inconclusive.

#### Anti-vascular endothelial growth factor (anti-VEGF)

Bevacizumab is a humanized monoclonal antibody against vascular endothelial growth factor (VEGF) activity that inhibits angiogenesis [141].

Its use as an adjuvant off-label therapy for recurrent conjunctival papilloma has recently been reported [142]. A 29-year-old patient with a history of 3 papilloma recurrences after surgical excision received a single dose of subconjunctival bevacizumab (0.2 ml, 25 mg/ml) intraoperatively after repeat excisional biopsy. No recurrence was noted after 37 months of follow-up. There are no other reports on the use of bevacizumab for the treatment of conjunctival papilloma in the literature.

In terms of side effects, the study above reported none. In addition, no local or systemic side effects were encountered with the use of anti-VEGF agents for the treatment of OSSN [143, 144]. However, the cost of anti-VEGF injections can limit its use [102]. Although potentially effective and safe, larger studies on the role of anti-VEGF in the management of conjunctival papilloma are needed.

#### Carbon dioxide (CO_2_) laser therapy

First used by otolaryngologists for tracheal and laryngeal papillomas, this approach consists of the use of infrared CO_2_ laser that generates temperatures of around 750 degrees Fahrenheit. The thermal damage not only precisely disrupts the surrounding 100 μm of tissue but is also thought to decrease the chances of viral seeding by inactivating HPV and sealing the lymphatic vessels [145, 146]. This treatment can provide a bloodless field of treatment, with minimal induced damage and scarring, which makes it well tolerated by patients [147].

The CO_2_ laser therapy was used in the 1980s concurrently with surgical excision for treatment of highly aggressive and recurrent conjunctival papillomas not responding to prior rounds of surgical excision, cryotherapy, chemotherapy, and immunotherapy [145–148]. In 75 cases of recurrent conjunctival papillomas that were vaporized with the CO_2_ laser and followed up for over 2 years, 2 recurrences were observed. Comparatively, the reported recurrence frequency with surgical excision alone was 20% [146].

#### Follow-up

During the medical treatment period, patients should be followed every 1 to 2 months to assess treatment response and identify possible adverse events. Patients treated surgically are usually seen 1 day, 1 week, 1 month, and every couple of months after surgery. Upon resolution, follow-up every 3 months during the first year, every 6 months during the second year, and yearly thereafter is generally indicated to rule out recurrence.

Recurrence frequencies after all treatments range from 3 to 27% [2, 3, 5, 48]. These rates are higher in the pediatric and adolescent population compared to adults [3]. In a study of 22 patients, a higher recurrence rate was seen with surgical excision alone (50%, 4 out of 8) when compared with surgical excision and adjuvant therapy (cryotherapy, CO_2_ laser or MMC) (7.1%, 1 out of 14) [48]. In another study of 73 patients, recurrence for excisional biopsy and cryotherapy was lower and noted in only 1 of the 61 patients treated [3]. These studies are very small to draw conclusions.

Other factors associated with higher recurrence include bulbar conjunctival location and corneal involvement [48]. No association between HPV 16 and 18, the high-risk types, and lesion recurrence after surgical excision have been noted [149]. Histopathologically, recurrent papillomas were found to exhibit moderate to severe epithelial dysplasia and higher mitotic activity (indicated by positive staining for Ki67 and p53) when compared with non-recurrent papillomas [149, 150].

For OSSN, it has been shown that the presence of microscopic disease at the lesion border may increase the risk of recurrence [123, 151]. This may also apply to papilloma; however, it has not been shown.

## Conclusions

Conjunctival papilloma is a benign epithelial tumor of the conjunctiva that can occur at any age in pediatric and adult patients. HR-OCT is a non-invasive diagnostic tool that can help identify lesions without the need for biopsy and assess treatment response. Papillomas can be managed medically, surgically, or with a combined approach depending on patient and tumor factors. Observation is also an option for small and asymptomatic tumors. Medical management is generally preferred given the theoretical lower risk of viral seeding. When used before surgery, medical therapy can debulk the lesion and minimize tissue excision. For papillomas that require surgical excision, a ‘no-touch’ technique along with cryotherapy and intraoperative MMC or IFN injection should be considered to reduce the risk of recurrences. Close surveillance is necessary to ensure timely detection and treatment of recurrences.

Despite the benign nature of the tumor, treatment can be challenging with the occurrence of medical treatment failures and recurrences after medical and surgical interventions. There is a paucity of published data with the use of topical chemotherapies as primary interventions, and overall from our experience as well as from the literature review, it seems that lesions with a higher degree of dysplasia tend to respond better to topical chemo- and immunotherapy. Lesions with no signs of dysplasia seem to be more recalcitrant and resistant to topical treatment. We hypothesize that this occurs due to a higher cell turnover in lesions with more dysplastic features, making them more susceptible to topical chemo- and immunotherapy.

Given that conjunctival papilloma is relatively rare, the majority of data is from reports and series, and no head-to-head comparisons between treatment approaches are yet available for this challenging condition. Hopefully the future will bring effective, new therapeutic options for patients with this condition.

## Data Availability

Not applicable.

## Abbreviations

5-FU: 5-fluorouracil
CIS: carcinoma in situ
CO2: carbon dioxide
DNCB: dinitrochlorobenzene
H2: histamine 2
HIV: human immunodeficiency virus
HPV: human papilloma virus
HR-OCT: high resolution optical coherence tomography
IFN: interferon
mm: millimeter
MMC: mitomycin C
ms: milliseconds
OSSN: ocular surface squamous neoplasia
PCR: polymerase chain reaction
Peg: pegylated
PV: papilloma virus
UBM: ultrasound biomicroscopy
UV: ultraviolet
